# The Statistical Determinants of the Speed of Motor Learning

**DOI:** 10.1371/journal.pcbi.1005023

**Published:** 2016-09-08

**Authors:** Kang He, You Liang, Farnaz Abdollahi, Moria Fisher Bittmann, Konrad Kording, Kunlin Wei

**Affiliations:** 1 School of Psychological and Cognitive Sciences, Peking University, Beijing, China; 2 Beijing Key Laboratory of Behavior and Mental Health, Beijing, China; 3 Peking-Tsinghua Center for Life Sciences, Beijing, China; 4 Rehabilitation Institute of Chicago, Chicago, Illinois, United States of America; 5 University of Wisconsin, Madison, Wisconsin, United States of America; 6 Northwestern University, Chicago, Illinois, United States of America; University College London, UNITED KINGDOM

## Abstract

It has recently been suggested that movement variability directly increases the speed of motor learning. Here we use computational modeling of motor adaptation to show that variability can have a broad range of effects on learning, both negative and positive. Experimentally, we also find contributing and decelerating effects. Lastly, through a meta-analysis of published papers, we verify that across a wide range of experiments, movement variability has no statistical relation with learning rate. While motor learning is a complex process that can be modeled, further research is needed to understand the relative importance of the involved factors.

## Author Summary

Variability is a fundamental component of our motor behaviors. It is caused by numerous factors, including sensory, planning, neuromuscular noise, as well as random external perturbations. Investigation of its underpinnings has been a driving force for numerous theoretical advances in motor control. Recently, it has been suggested that initial motor variability can promote the speed of motor learning. We first demonstrate with a series of simulations of a common learning model that different factors leading to increased variability can affect learning rate in completely different directions, instead of merely the positive trend as claimed. Second, we present experimental evidence that sensory uncertainty, which affects motor variability, instead of variability *per se*, determines learning speed during trial-by-trial random perturbations. Third, we present results from a meta-analysis of published studies that show the same lack of positive correlation. We conclude that motor learning is not generally facilitated by initial motor variability. Instead, their relationship should be investigated by considering the factors that affect variability in a task-specific manner.

## Introduction

We can never produce exactly the same movement twice. Producing one continuous movement is invariably overlaid with fluctuations. Motor variability is defined as the variation of performance across repetitions or continuous performance of the same task. It has been demonstrated in various aspects of movements, starting with movement outcomes such as errors in reaching [1] or throwing [2,3], variance in force production [4,5] and body sway during quiet standing and walking [6,7], to the spread of movement trajectories [8,9] and variable coordination between effectors [10].

Motor variability is central to motor control and coordination. It was probably Bernstein who raised awareness of the degree-of-freedom problem when he first observed that even a skilled blacksmith cannot consistently hit the anvil due to varying trajectories of the hammer across repeated attempts [11]. A large body of research has examined the temporal and spatial patterns of motor variability as a window to elucidate the mechanisms underlying control and coordination of movements [12,13]. For instance, when controlling multiple degrees of freedom simultaneously, the motor system appears to reduce the variability in task-relevant dimensions, while leaving it unregulated in others. This variability pattern has been widely regarded as a signature of control [14–18]. The fact that motor variability is differentially regulated by feedback is also regarded as critical evidence for optimal feedback control [8]. The inverse of motor variability, i.e., regularity in movements or stability of task performance, has also been systematically investigated in the area of motor control. For instance, straightness of reaching trajectory has been proposed to reflect that the motor system aims to minimize the impact of motor noise on the movement outcome [19]. Variability and stability of task performance have been used to dissociate between error correction and self-stabilizing processes [20]. Thus, motor variability poses as a fundamental problem for neural control of movements and has received significant attention.

Studies on the relation of motor variability with learning are comparatively sparse, except those that demonstrate that reduction of variability is a fundamental characteristic of learning [21]. In a series of studies, Sternad and colleagues went one step further and decomposed motor variability into different functional components to demonstrate that skill acquisition is a multi-stage process of finding a stable solution where the detrimental effect of inherent neuromotor noise onto performance is reduced [3,22–25]. Interestingly, a recent study reported that learners’ initial variability is positively correlated with their rate of improvement for both reinforcement learning and motor adaptation [26]. This finding is consistent with the frequently claimed positive effect of initial exploration in reinforcement learning [27].

As theoretically elegant this result may be, this facilitatory effect of motor variability has not been reported before and begs the question how universal this finding may be. That variability can be beneficial has been shown in the realm of perception: variability in sensory signals can enhance perception and improve related sensorimotor tasks due to stochastic resonance [28–32]. However, this is not a universal mechanism, as this facilitatory effect is limited to detection of sub-threshold sensory signals that would go undetected without extra noise. Similarly, we know that motor learning is highly diverse, ranging from motor adaptation to acquisition of complex motor skills [21,25,33]. It remains unknown if initial variability generally facilitates motor learning.

More importantly, motor variability itself stems from multiple sources, including execution noise in motor commands [19,34,35], observation noise or uncertainty inherent in the sensory system [36,37], inaccurate estimates of external parameters [38], noise in motor planning [39], and the disturbances in the external world [40]. These factors could simultaneously affect the learning rate and motor variability in distinct ways, leading to positive, negative or zero relations between them. We hypothesize that motor variability is correlated to learning rate in a task-specific way, depending on which factor predominantly affects task performance.

To test this hypothesis, we performed computer simulations of error-based learning with a widely accepted optimal learner model. The simulation highlights the fact that multiple factors can simultaneously affect variability and learning speed in distinct ways. We also designed a series of four experiments where motor variability varied due to execution noise. We applied visual perturbation on a trial-by-trial basis to highlight the role of visual feedback. We found that adaptation rate was correlated to variability in completely different ways across tasks, but that their relations appeared consistent with predictions based on sensory uncertainty of error feedback. We also presented a new meta-analysis to show a lack of relation between variability and learning rate across a broad range of previously published data.

## Methods

### Ethics statement

All participants were näive to the purpose of the study, signed an institution-approved consent form, and were paid to participate. All experimental procedures were approved by the Institutional Review Board of Peking University.

### Simulation of an optimal learner model

Previous motor adaptation studies have found that people appear to efficiently learn from error feedback by taking uncertainty into consideration, consistent with predictions of Bayesian statistics [e.g., 41]. Here we opt to use a Kalman filter, a widely used model of motor learning, to construct an optimal learner model for three reasons. First, as a specific form of Bayesian model, the Kalman filter has been frequently used for modeling trial-by-trial learning and its predictions match well with actual human behavior [37,40,42]. Second, it enables us to measure the variance of performance since it updates state estimates on trial-by-trial basis [43]. Third, we can systematically modify model parameters and examine the resulting relation between motor variance and learning rate.

The Kalman filter makes predictions of the state on trial-by-trial basis [34,40]:

$${\hat{x}}_{k|k-1}=A{\hat{x}}_{k-1|k-1}$$ (1)

$$P_{k|k-1}=AP_{k-1|k-1}A^{T}+Q$$ (2)

where $\hat{x}$ is the state estimate of the body and the world (in our case the rotation of the reaching direction), *A* is the transition matrix from one trial to the next. ${\hat{x}}_{k|k-1}$ denotes a priori (predicted) state estimate before receiving the feedback in the *k*-th trial. For example, it can be the intended movement direction before the *k*-th reach in a visuomotor rotation paradigm. *P*_*k*−1|*k*−1_ is the estimate covariance in trial k-1 and its prediction *P*_*k*|*k*−1_ is made with process noise following ∼*N*(0,*Q*). For each trial, the Kalman model also updates its estimates:

$$y_{k}=Z_{k}-H{\hat{x}}_{k|k-1}$$ (3)

$$S_{k}=HP_{k|k-1}H^{T}+R$$ (4)

$$K_{k}=P_{k|k-1}H^{T}S_{k}^{-1}$$ (5)

$${\hat{x}}_{k|k}={\hat{x}}_{k|k-1}+K_{k}y_{k}$$ (6)

$$P_{k|k}=(I-K_{k}H)P_{k|k-1}$$ (7)

where *Z*_*k*_ is the actual feedback, *H* is the observation matrix that maps the state estimate to the observable state and here it is set as [11] for the two-dimensional states (see the definition of dimension below), *y*_*k*_ is the error signal (or innovation) that drives the learning, *S*_*k*_ is the covariance matrix for *y*_*k*_ and it is updated in each trial with observation noise following ∼*N*(0,*R*). As shown in Eq 6, the error is partially corrected according to a Kalman gain *K*_*k*_, which is an optimal learning percentage that is determined by taking into consideration of process noise and observation noise. Thus, we can obtain the a posteriori state estimate ${\hat{x}}_{k|k}$ after observing the error feedback in trial k. Lastly, the estimate covariance is updated in each trial (Eq 7).

Since motor learning has been shown to involve multiple time scales [10,44–46], our model takes different time scales into consideration. For simplicity, we only model the learning state with a fast process and a slow process, similar to the two-state model proposed by Smith and colleagues [44]. Thus, the state estimate has two dimensions but the model output is a sum of these two hidden states. The model parameters A and Q are diagonal matrices. We set initial parameters as $A=\left[\begin{array}{cc} 0.998 & 0\\ 0 & 0.75 \end{array}\right]$, $Q=\left[\begin{array}{cc} 1.46 & 0\\ 0 & 146 \end{array}\right]\times 10^{-7}$ and *R* = 3.0 × 10^−4^. The only constraint to choose these initial parameter values is to make the model simulation close to human performance. For example, movement variability, the extent of achieved learning and learning rate should be appropriate as compared to actual performance in motor adaptation paradigms such as adapting to a visuomotor rotation and to force fields. With our default parameter values, movement variability (as quantified by standard deviation over unperturbed trials) amounts to about 6% of the movement amplitude, about 92% of a constant perturbation is compensated after learning asymptotes, and half of initial error is corrected after about 35 trials (see the description below about the simulation of motor adaptation). We also confirm that the simulated effects are robust when we vary the system parameters by a factor of 10 (see Results).

The basic simulation procedure goes as follows. Initially, we simulate a sequence of 10000 trials with a linear dynamic system that is identical to the Kalman filter model but without the recursive update based on feedback. This model is initialized to generate an output of 0 (arbitrary unit, a.u.) and it iterates with the same parameters (*A*, *Q* and *R*) as the Kalman model. This generates a sequence of the observed state *Z*_*k*_ (Eq 3), which is essentially a steady-state sequence (around 0) and affected by state noise and observation noise. Then, using *Z*_*k*_ as the actual observed feedback, we simulate the behavior of our Kalman model for 10000 trials. The baseline variance is calculated as the standard deviation of movement errors over all the trials except the initial 1000 trials to exclude initial transients. We then perturb the model by subtracting a constant 0.3 to the observation, simulating a step-wise perturbation typically employed in motor adaptation studies (see Results). The Kalman model converges to the perturbed state in an exponential fashion and thus generates an error-based learning curve. We fit an exponential function *y* = *a* + *be*^−*ct*^ to this learning curve where *c* is the learning rate with a unit of trial^-1^. Thus, we can study the behaviors of an optimal learner model with different parameter settings. To examine the effect of observation noise and process noise, we systematically vary *Q*, and *R* by multiplying their initial values with a scaling factor from 1 to 10. To examine the effect of deviations from optimal learning, we artificially change the feedback gain (i.e. the Kalman gain) by multiplying it with a scaling factor from [1/16 1/8 1/4 1 4 8 16]. Thus, the trial-by-trial feedback gain deviates from the optimal values in both increasing and decreasing directions. It is unchanged with the scaling factor of 1. Lastly, to examine the effect of the relative contribution of the two time-scale states, we selectively multiply the fast-state noise in *Q* by a scaling factor from 1 to 10. This effectively amplifies the contribution of the fast state. Based on simulations with these parameter modulations, we compute the corresponding baseline variability and learning rates and examine their relations.

### Experiments

We recruited a total of 90 subjects, 20 subjects for each of the 4 experiments and 10 for a control experiment. For all experiments, the order of conditions was counterbalanced between subjects.

We chose four tasks, including reaching movements towards different directions (Exp1), towards the same direction but with different distances (Exp2), towards the same target but with visual perturbations in different directions (Exp3), and isometric force production with different force magnitudes (Exp4). All experiments involve two conditions with different levels of variability that have been mainly attributed to motor noise [1,35].

**Fig 1:**
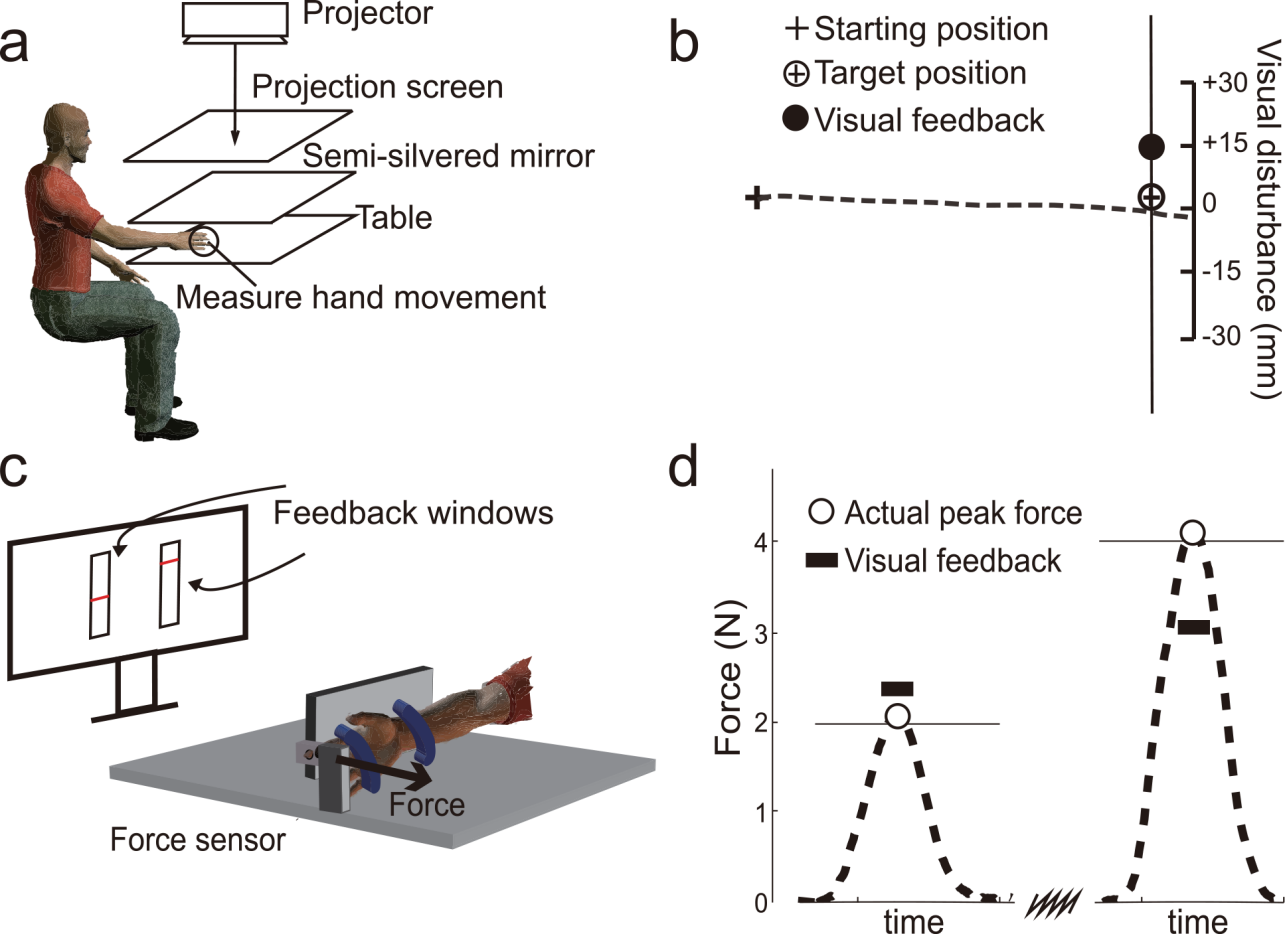
Experimental setup and exemplary movement/force trajectories. a) An illustration of the experimental setup used for Experiments 1–3. Vision of the hand and the arm is blocked by a semi-silvered mirror. b) A typical movement trajectory in Exp1 (shown as a dashed line) and its associated endpoint feedback. The visual feedback is displayed briefly in the direction perpendicular to the reaching direction. As shown in the illustration, this means that the visual feedback only appears on the line (invisible to the subject) passing through the target. In some trials, this endpoint feedback is perturbed along this direction with a randomly assigned magnitude. The target shown here is in the 0° direction. c) An illustration of the experimental setup in Exp4. The force exerted by the right index finger is measured by a force transducer mounted on a fixed handle. The magnitudes of the two target forces are shown as two red lines displayed on a monitor. d) Typical force trajectories in Exp4, shown as dashed lines. The target forces are shown as solid horizontal lines. As an endpoint feedback, the peak force is briefly shown to the subject and it is occasionally perturbed.

For Exp1-3, subjects made planar, center-out hand reaches to visual targets projected on a table top (Fig 1A). Their actual hand movements were concealed by a projection screen. The fingertip location was measured throughout the experiment by an attached infrared marker (Codamotion, Charnwood Dynamics, UK; sampling rate ~200 Hz). Data acquisition and screen display were controlled by a customized Matlab program (Matlab 2009b; MathWorks, Natick, MA). Visual feedback of the hand, as a cursor, was only briefly shown at the beginning and at the end of a reaching movement [47]. We used a motor adaptation paradigm to visually perturb the endpoint feedback on trial-by-trial basis (Fig 1B). The learning to a single perturbation was assessed in a block fashion. Within a trial block, subjects reached with veridical visual feedback for either one, two or three trials (null trials), followed by a perturbation trial. In this trial, the hand cursor deviated from the actual hand position and this perturbation typically made subjects to deviate their hand movement in the next trial. The next trial following the perturbation trial was the last trial in a trial block and it had no endpoint feedback. This was the only trial that had no endpoint feedback within a trial block. The amount of hand deviation in this test trial signified the learning rate. For trial-by-trial learning, how much an imposed perturbation is corrected in the next trial is indicative of learning rate [37]. After this test trial, a new block began. The size and direction of the perturbation for each block were randomly chosen from a pre-defined set of values. The specifics for Exp1-3 are described below.

In Exp1, subjects repetitively reached to a target, which was displayed as a 10-pixel-diameter disc that was 150 mm away from the starting position. There were two possible targets in two different directions. Defining the rightward direction as 0° and the straight-ahead direction as 90°, one target was in the 10° direction and the other in the 150° direction. The amount of learning associated with the two targets was examined in two separate sessions. For each session, subjects first familiarized themselves with the task by performing 20 trials with veridical visual feedback. Once their hand moved beyond the target, a 7-pixel-diameter cursor was displayed for 300ms to indicate the hand location along the orthogonal direction of the reach (Fig 1B). A warning sound was played when the movement time exceeded 500ms. Only 2.8%, 2.1%, 0.5% and 0% of total trials had movement time larger than 500ms in Exp 1, 2, 3, and 4, respectively. After the familiarization phase, we measured their baseline variability by asking them to perform 40 trials with veridical feedback (the baseline phase). Specifically, the standard deviation (SD) of the endpoint scatter in the orthogonal direction was computed for each subject. The 150° target was associated with larger endpoint variance as compared to the 10° target [1]. In the subsequent adaptation phase, their learning of visual perturbation was tested with blocks of trials as described above. For the perturbation trial in a trial block, the endpoint feedback was not veridical any more. Instead, a spatial deviation was applied along the orthogonal direction with a magnitude randomly selected from [0,±15,±30]mm. We defined the visual perturbations that deviate the endpoint feedback away from the body as the positive ones and the perturbations towards the body as the negative ones (same below). Each perturbation size was tested for 10 times (10 blocks of trials) with three 3-trial blocks, four 4-trial blocks and three 5-trial blocks. The order of blocks and perturbation sizes were fully randomized within a target session. These arrangements minimized the possibility that subjects anticipated the perturbation trial and acted proactively. The block size did not affect the learning rate as confirmed by post-hoc analysis. By regressing the hand deviation in the test trial against the magnitude of the preceding perturbation (see Results), we quantified the learning rate by the regression slope [37]. For each target, there were 20, 40 and 200 trials for the familiarization, baseline and adaptation phases, respectively. The total number of trials was 520.

In Exp2, subjects reached to a 0° target which was either 75mm or 150mm away. Previous studies have found that the larger the reaching distance the larger the endpoint variance [48]. The experimental setup and procedures were identical to those of Exp1. Again, visual perturbations were applied in the orthogonal direction and we measured subjects’ baseline variability and learning rate.

In Exp3, subjects reached to a 0° target which was 6cm away and the direction of visual perturbation was manipulated across trials. In contrast to Exp1 and 2, subjects were required to stop at the target as accurately as possible. The end of a movement was defined as the time when the movement speed dropped below 5mm/s. The visual feedback was perturbed either in the movement direction or in its orthogonal direction. For hand reaching, the endpoint variability is higher in the movement direction than in the orthogonal direction [1]. Trials from these two conditions were interspersed in a random fashion. On each trial, the perturbation magnitude was randomly selected from [0,±5,±10]mm. We chose these relatively small perturbation sizes as learning to large perturbations was not linearly proportional for the reach distance examined here [49]. The familiarization, baseline and adaptation phases contained 60, 40 and 400 trials, respectively. This resulted in a total of 500 trials.

In Exp4, subjects produced an isometric force pulse against a mounted force transducer (Fig 1C; ATI, model Nano 17, resolution 0.0035N). Their force magnitude could be shown as an 8-pixel-long bar on a computer monitor whose height was proportional to the force magnitude. Subjects were required to produce a peak force as accurately as possible to a target force (either 2N or 4N). Each target force was shown as a horizontal line at the vertical center of a feedback window. The feedback window for the 2N target force was on the left half of the screen and the window for the 4N target was on the right. The target appeared at the start of each trial, triggering the subject to briefly press against the force transducer. Similar to Exp1-3, the cursor was not shown during the action and only the end result (the peak force) was briefly shown as a short horizontal bar, which remained visible for 300ms (Fig 1D). The subject was instructed to use the horizontal bar to “hit” the target force as accurately as possible. Note the vertical position of this visual representation of force was fully determined by the force magnitude. The scaling between the bar displacement and the force magnitude remained consistent across two conditions. After this endpoint feedback disappeared, the subject relaxed for 3000ms until the next trial started. The experimental procedure was nearly identical to Exp1. Two force conditions were tested in separate sessions. Participants first familiarized themselves with the task for 60 trials. In the baseline phase (40 trials), we quantified their individual motor variability in terms of SD of peak force. People typically exhibit larger variance in peak force when producing a larger force [4,50]. In the adaptation phase the visual endpoint feedback was perturbed from its veridical height with a magnitude randomly selected from [0,±0.3,±0.6]N. Subjects would increase or decrease their force following a perturbation trial. Each perturbation size was tested for 10 times, including three 4-trial blocks, four 5-trial blocks and three 6-trial blocks. The total number of trials was 700.

We also performed a control experiment to measure visual uncertainty of locating an object at different depths in the setting of Exp1. Subjects were asked to discriminate the location differences of cursors at the two target locations in Exp1. They performed a two-alternative forced choice task (2AFC) without movements. The task was to judge the relative position of two sequentially-presented cursors, which were identical to the endpoint feedback cursor used in Exp1. During each trial, the subject first fixated at a fixation cross in the middle of the workspace for 500ms. After the fixation cross disappeared, the two cursors were subsequently displayed for 750ms, with an inter-stimulus interval of 1000ms. The reference cursor was always displayed at the target position (the 10° or 150° target). A test cursor was displaced from the reference cursor along the orthogonal direction, with a magnitude randomly selected from [0,±0.7,±1.4,±2.1]mm. These two target positions were tested in separate sessions. The order of the two sessions was counterbalanced between subjects. For the 10° session, the subject was asked to judge whether the second stimulus located above or down to the first stimulus; for the 150° session, the task was to judge whether the second stimulus located down-left or right-above to the first stimulus. The subject responded by pressing the left or right arrow key on a keyboard. No correct-answer feedback was given. The order of the reference stimulus and target stimulus was randomized across trials. Each reference-target pair was presented 20 times and their order was fully randomized. The sensory uncertainty was quantified as σ in a probit function fitted from the psychometric curve. The fitting was performed by using fminsearch algorithm in Matlab (R13, Mathworks Natick, USA).

### Meta-analysis of previous motor adaptation studies

We selected 5 studies from the DREAM project, a collaborative datasets from published, behavioral studies with reaching experiments. The inclusion criterion was that the experiment must have a block-based design with a session of baseline performance and a session of motor learning. Five studies were selected including adaptation to visuomotor rotation [51], visuomotor gain [52], and velocity-dependent force fields [53–55]. The numbers of subjects are 16, 37, 36, 30 and 13, respectively. The total number is 132.

We computed the baseline variability with performance measures that are typically reported for these perturbations studies. For velocity-dependent force field, the performance measure was the maximum lateral deviation of the reaching trajectory. For visuomotor rotation, it was the direction error from the desired movement direction. For visuomotor gain, it was the distance error from the desired movement distance. For Ostry et al. (2010) [53], Vahdat et al. (2011) [55] and Wei et al. (2014) [52], the baseline variability was computed as standard deviation for the last 50 trials of a baseline session, while for Mattar and Ostry (2010) [54] we took the last 20 trials given limited number of trials. For the same reason for Fernandes et al. (2012) [51], we used the last 15 baseline trials before the perturbation session.

To derive the learning rate, we fit learning curves (error as a function of trials) with an exponential function (*y* = *a* + *be*^−*ct*^) where time constants *c* signifying the learning rate of each individual subject. For fitting these learning data of varying rates, we used 150, 150, 200, 240, 30, and 60 trials from Mattar & Ostry, Ostry et al., Vahdat et al., Fernandes et al., Exp1 of Wei et al. and Exp2 of Wei et al., respectively. 123 out of 132 fits were significant with an average r^2^ of .36±.02. To pool over all data sets, we normalized baseline variability and learning rates by computing their respective z-scores. As some data points appeared to be outliers, we also performed non-parametric correlation analysis.

## Results

### Model simulation

**Fig 2:**
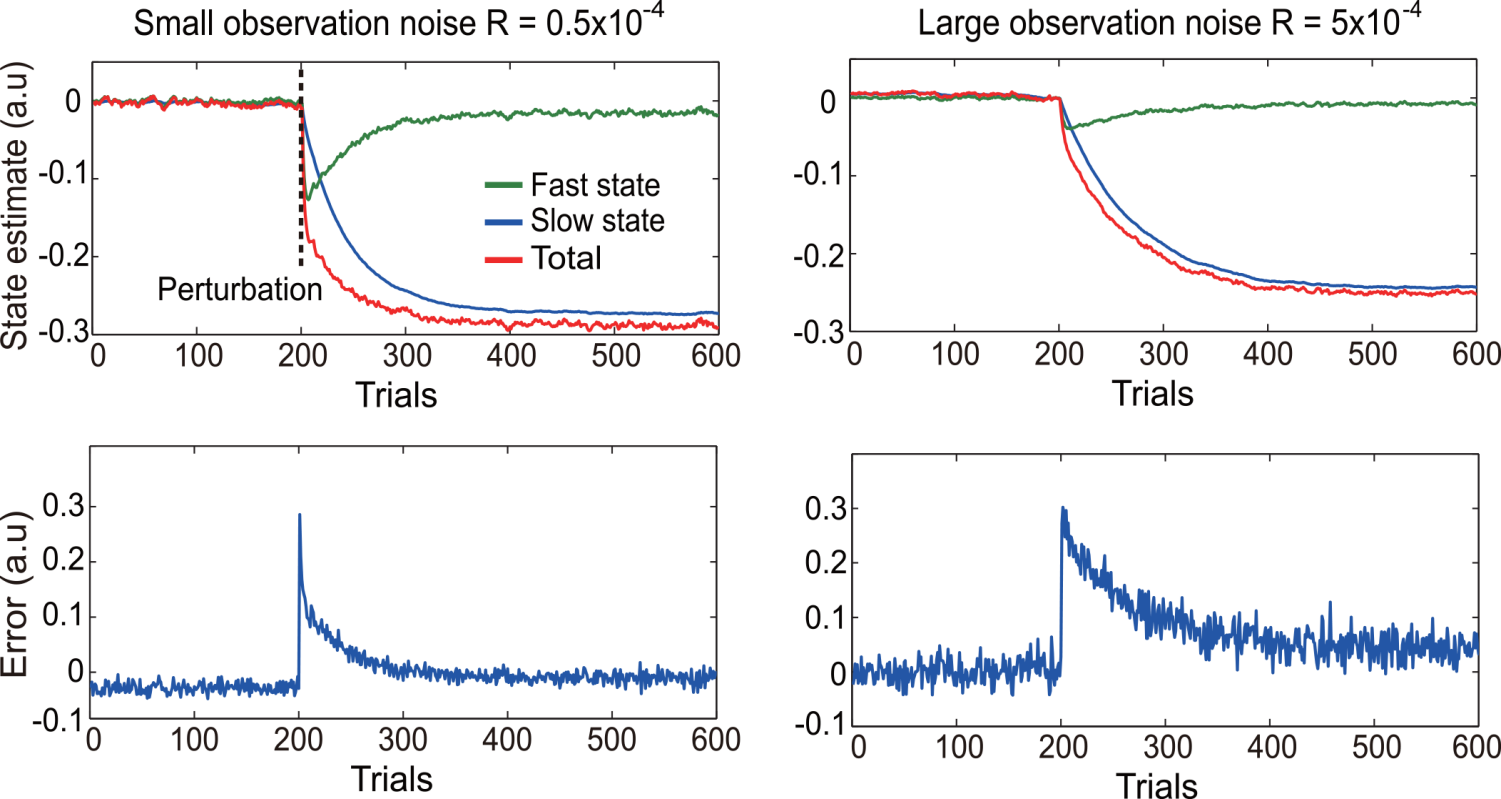
Exemplary model simulations when observation noise (R) is varied. The left two panels are for a small *R* value and the right two panels are for a large *R* value. Other model parameters remain the same in the simulations. The two top panels display the changes of the state variables as a function of trials. The two hidden states, a slow and a fast component, and their sum are plotted separately. The perturbation is applied at the 201^th^ trial. The two bottom panels display the corresponding movement error, i.e., the difference between the model estimate and the actual feedback, as a function of trials.

The Kalman model changes its state once the perturbation is applied (Fig 2). As expected, the fast component changes faster than the slow component. The difference between the predicted state and the actual feedback is the movement error (two bottom panels). Before the perturbation is applied, the error fluctuates within a small range where baseline variability is computed. It abruptly increases upon perturbation and decreases exponentially afterwards. The learning rate can be estimated by fitting an exponential function to the learning curve. In the exemplary simulations, increasing the observation noise by a factor of 10 leads to more baseline variability and slower learning (the left panels vs. the right panels).

**Fig 3:**
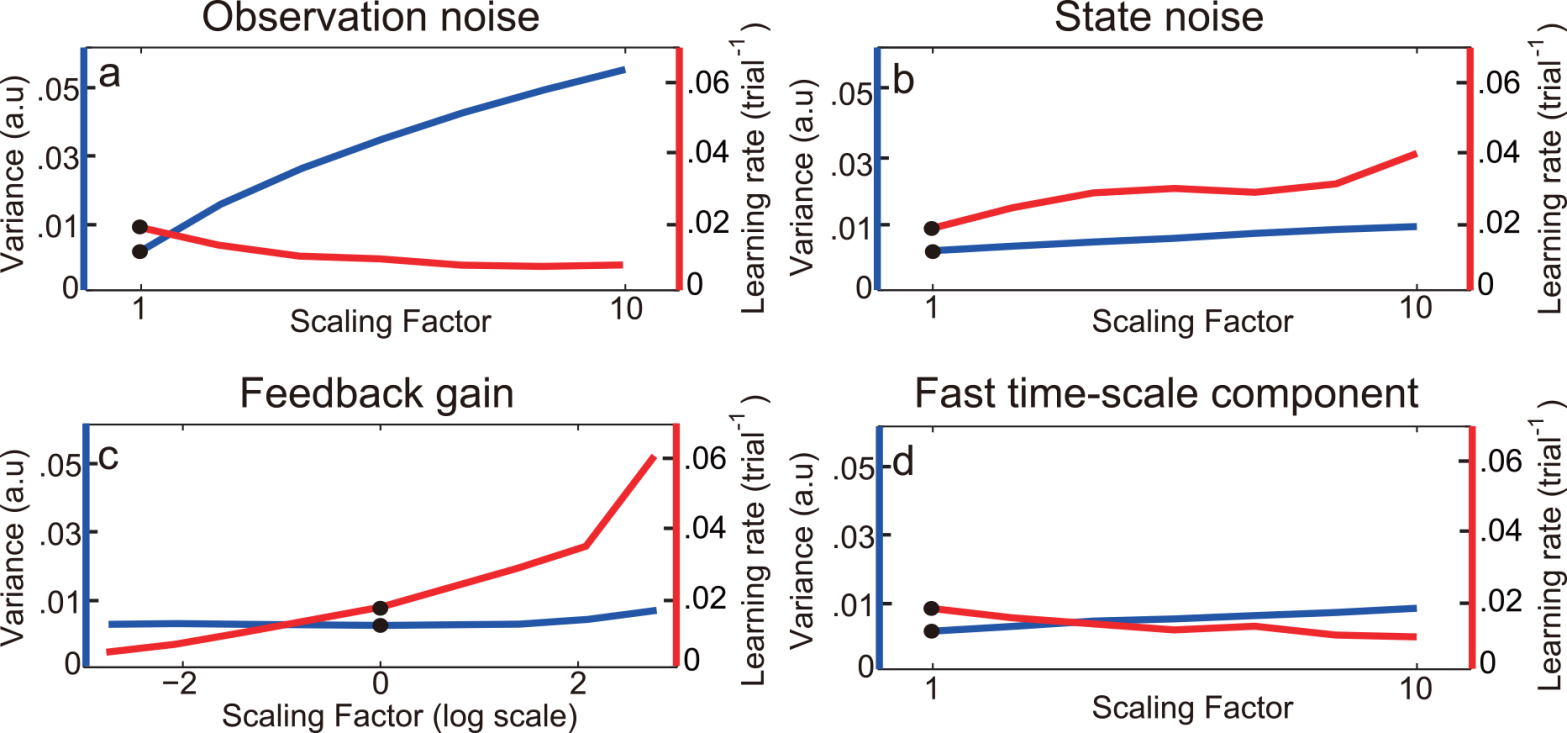
Simulation results from an optimal learner model. Varying the levels of observation noise (a), process noise (b), feedback gain (c), and magnitude of the fast process (d) can lead to different relations between motor variance and learning rate. Their levels are manipulated by multiplying a scaling factor. For example, the feedback gain (the original Kalman gain, panel c) is modified or left unchanged. The black dots highlight the simulation conditions with the same parameter setting across panels where no scaling is applied. Variability has an arbitrary unit and learning rate has a unit of trial^-1^. Note panel c is shown in a log scale.

With the common assumption of an optimal learner, a simple simulation can highlight a broad set of possible relations between variability and learning rate (Fig 3). (1) If there is more observation noise then variability will be higher as the brain does not know what is veridical; but learning will be slower because the new information is not so useful [37]. Thus, variability and learning rate go in opposite directions with increasing observation noise (Fig 3A). (2) With more process noise, we simulate the situation that the body or the world is changing more rapidly (Fig 3B). This will lead to both more variability, as a direct result of the changes, and faster learning, which compensates for those changes [40]. Thus, variability and learning rate change in the same direction with increasing process noise. (3) If the brain uses a learning rate that is different from that of an optimal learner, it will increase variability [34]. As shown in the simulation, the minimum variability is obtained with a standard Kalman gain with a scaling factor of 1 (Fig 3C). Any deviation from this scaling factor, effectively reducing or increasing the feedback gain, leads to more variability (not apparent in the graph due to the scale of the plot). Interestingly, the learning rate increases within the range of our simulation. (4) Learning consists of components of distinct time scales. By increasing the relative magnitude of the fast component, we find that variability increases, possibly due to the fast changes of the system (Fig 3D). Also, the relative contribution of the fast component in state estimates increases. As a result, when a constant perturbation is applied, the optimal learner model learns slower. This is reasonable since the perturbation is a long-term change but the state estimation is more dominated by the fast component when its relative magnitude increases. This mismatch makes the learning slower. In sum, the relation between variability and learning rate varies widely, depending on what factor is modulated. Thus, we expect that if one factor dominantly affects the performance for a specific task, this factor will largely determine the relation between variability and learning rate. In other words, the relation between variability and learning rate should be task-specific.

Of the aforementioned computational factors that simultaneously affect motor variability and learning rate, some of them predict a positive correlation between variability and learning rate (e.g. prediction 2) and some of them predict the opposite trend. For example, more sensory uncertainty leads to more variability but slower learning (prediction 1). Sensory uncertainty is critical for trial-by-trial adaptation investigated in the present study since learning here hinges upon a single visual perturbation whose uncertainty level is modulated across conditions.

### Experimental results

**Fig 4:**
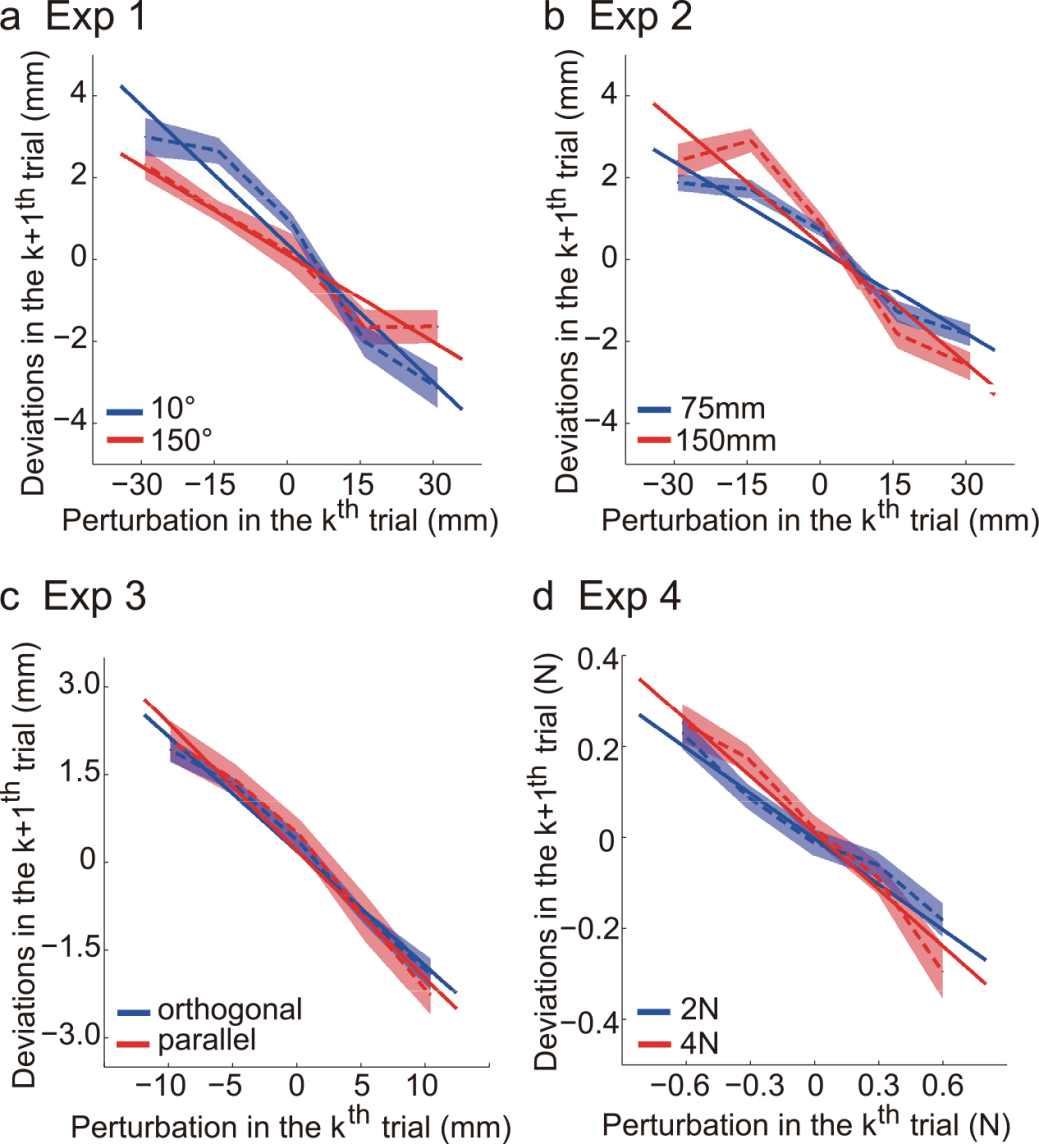
Average adaptation data across subjects from Exp1-4. a) In Exp1, the hand deviates in the opposite direction of the perturbation in the previous trial. The negative slope indicates the learning rate; reaching to the 150° target is associated with faster learning. b) In Exp2, reaching to the 150mm target is associated with faster learning. c) In Exp3, adaptation to perturbations in the movement direction (parallel) and in the orthogonal direction have similar learning rates. d) In Exp4, the peak force changes its magnitude following a visual perturbation. The two conditions with a 2N force production and a 4N force production are associated with similar learning rates.

Across four experiments we ask how variability relates to learning speed. After a visual perturbation was applied, subjects typically acted in the opposite direction of the perturbation, shown as either a hand deviation in the next reach or a force deviation in the next force production (Fig 4). This adaptation was linearly proportional to perturbation size (except for Exp2), thus the learning rate could be quantified as the linear slope of their relation.

**Fig 5:**
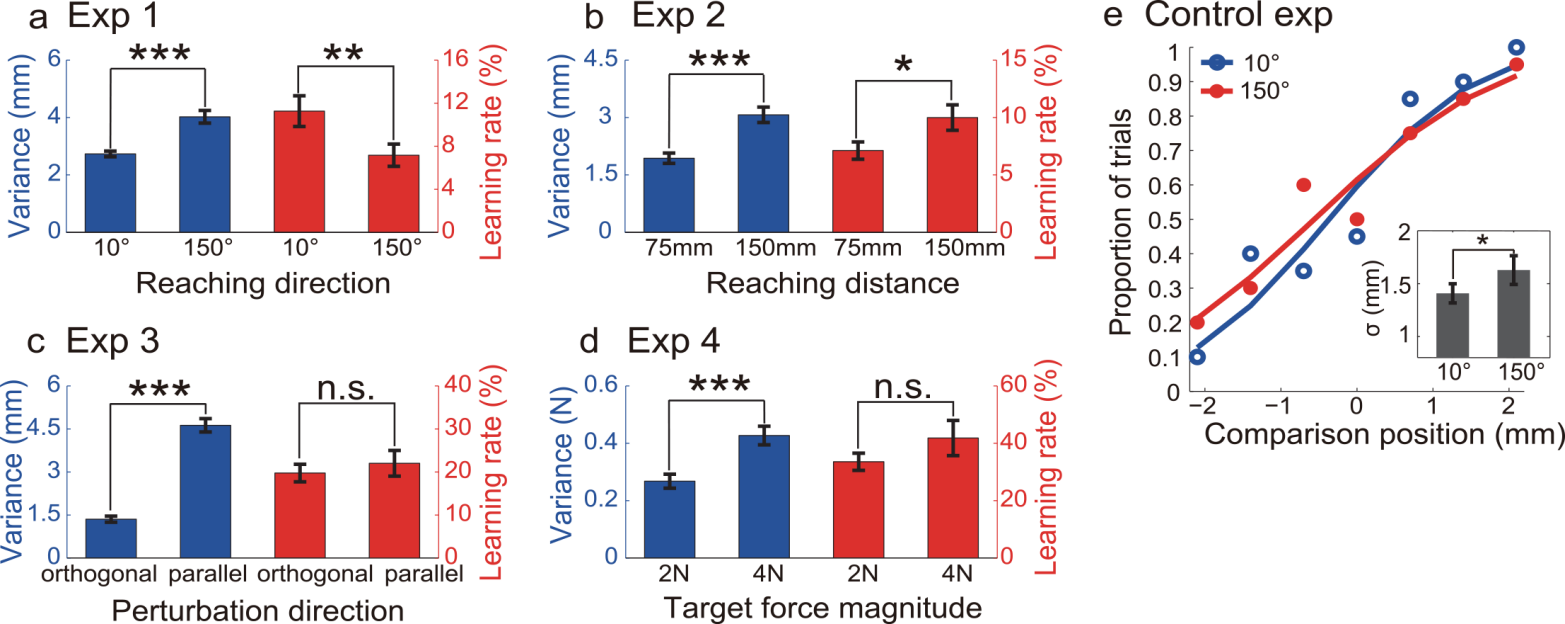
Average results from Exp1-4 and Control experiment. a) Reaching to the 150° target is associated with more endpoint variability than reaching to the 10° target. This larger variability is associated with slower learning. b) Reaching to a more distant target (150mm) is associated with more endpoint variability than to a nearer target (75mm). This larger variability is associated with faster learning. c) Variability is higher in the movement direction (parallel) than in the orthogonal direction, but the learning rates in these two directions are similar. d) Isometric force production of a larger magnitude (4N) is associated with more variability than that of a smaller magnitude (2N). However, the learning rates are similar. e) Visual discrimination performance from a typical subject in the control experiment based on 2AFC. The inset denotes the group average of σ, obtained by fitting the psychometrical curves of individual performance, as a function of target locations. The target with more depth (the 150° target) has higher visual uncertainty than the target with less depth (the 10° target). Blue bars stand for learning rates and red bars stand for variance (mean±SEM). *, ** and *** denote *p* < .05, < .01 and < .001, respectively.

We measured baseline motor variability for each individual subject before they were exposed to visual perturbations. As expected, the two conditions in each experiment yielded significant differences in motor variability (Fig 5). In Exp1, reaching to a target with more depth (150° vs. 10°) was associated with more endpoint variability (t_19_ = 5.54, *p* < .0001). The SDs of endpoint scatter in the orthogonal direction of movement were 4.02±0.22mm (mean±SEM) and 2.73±0.10mm for the 150° and 10° targets, respectively (Fig 5A). In Exp2, reaching to a more distant target (150mm vs. 75mm) was associated with more motor variability (t_19_ = 4.88, *p* < .0001). The SDs were 3.07±0.20mm and 1.94±0.14mm for the 150mm and 75mm targets, respectively (Fig 5B). In Exp3, when reaching to a single target, the SD of endpoint scatter was larger in the movement direction than in the orthogonal direction (t_19_ = 16.23, *p* < .0001). The SDs were 4.63±0.23mm and 1.36±0.11mm for these two directions, respectively (Fig 5C). In Exp4, the larger force production led to more force variability than the smaller force production (t_19_ = 6.178, *p* < .0001). The SDs were 0.43±0.03N and 0.27±0.02N for the 4N and 2N conditions, respectively (Fig 5D). In sum, baseline motor variability was distinctively different between movement conditions in all four designed tasks.

As shown above, the visual perturbations successfully induced adaptation (Fig 4). The learning rate, quantified as the regression slope, exhibited significant but diverse patterns between variability conditions (Fig 5). In Exp1, learning rate was significantly higher when reaching to the 10°target than when reaching to the 150° target (11.3±1.4% and 7.2±1.0%, t_19_ = -2.99, *p* < .007). In Exp2, learning rate was significantly higher when reaching to the distant target than when reaching to the near target (7.1±0.8% and 10.0±1.1%, t_19_ = 2.73, *p* < .013). In Exp3, the orthogonal-direction perturbation and the parallel-direction perturbation induced similar learning rates (19.7±2.0% and 22.0±3.0%, t_19_ = 0.634, *p* = .534). In Exp4, visual perturbations in the 2N condition and in the 4N condition were associated with similar learning rates (33.5±3.0% and 41.8±6.1%, t_19_ = 1.477, *p* = .156). Hence, the larger motor variability was associated with slower (Exp1), faster (Exp2) and similar learning rates (Exp3&4). These within-subject comparisons thus indicate that there was no clear association between variability and learning rate.

The relation between perturbation and hand deviation appeared to be nonlinear for Exp2 (Fig 4B) which might confound our conclusion. We re-analyzed the data without the two largest perturbations (±30mm) that might lead to deviations from straightness. After excluding the data from these extreme perturbations, the learning rates increased to 10.2±1.4% and 16.0±1.7% for the 75mm and the 150mm conditions, respectively. Importantly, the learning rate difference between conditions became more significant (changing from previous *p* < .013 to *p* < .003). Thus, the finding in Exp2 was not affected by possible deviations from straightness.

We used the linear slope to quantify learning rate (Fig 4); an alternative but more generic way is using two-way ANOVAs to examine the interaction effect between perturbation size and variability condition. These perturbation (5)×condition (2) repeated-measure ANOVAs confirmed the above slope-based findings. The interaction was highly significant with F(4,76) = 4.58, *p* < .0023 and F(4,76) = 4.55, *p* < .0024 for Exp1 and Exp2, respectively. The interaction was not significant with F(4,76) = 0.29, *p* = .88 and F(4,76) = 1.68, *p* = .16 for Exp3 and Exp4, respectively. As such, these results were in line with our standard analysis.

The control experiment measured the visual uncertainty of locating a cursor with different depths. Specifically, this visual uncertainty was evaluated at two target locations in Exp1. The σs obtained by fitting a probit function to the psychometric function were 1.41±0.09mm and 1.63±0.14mm for the 10° and 150° targets, respectively. Thus, locating a cursor in the 150° target condition was associated with more sensory uncertainty than in the 10° target condition (t_9_ = 2.57, *p* < .05).

**Fig 6:**
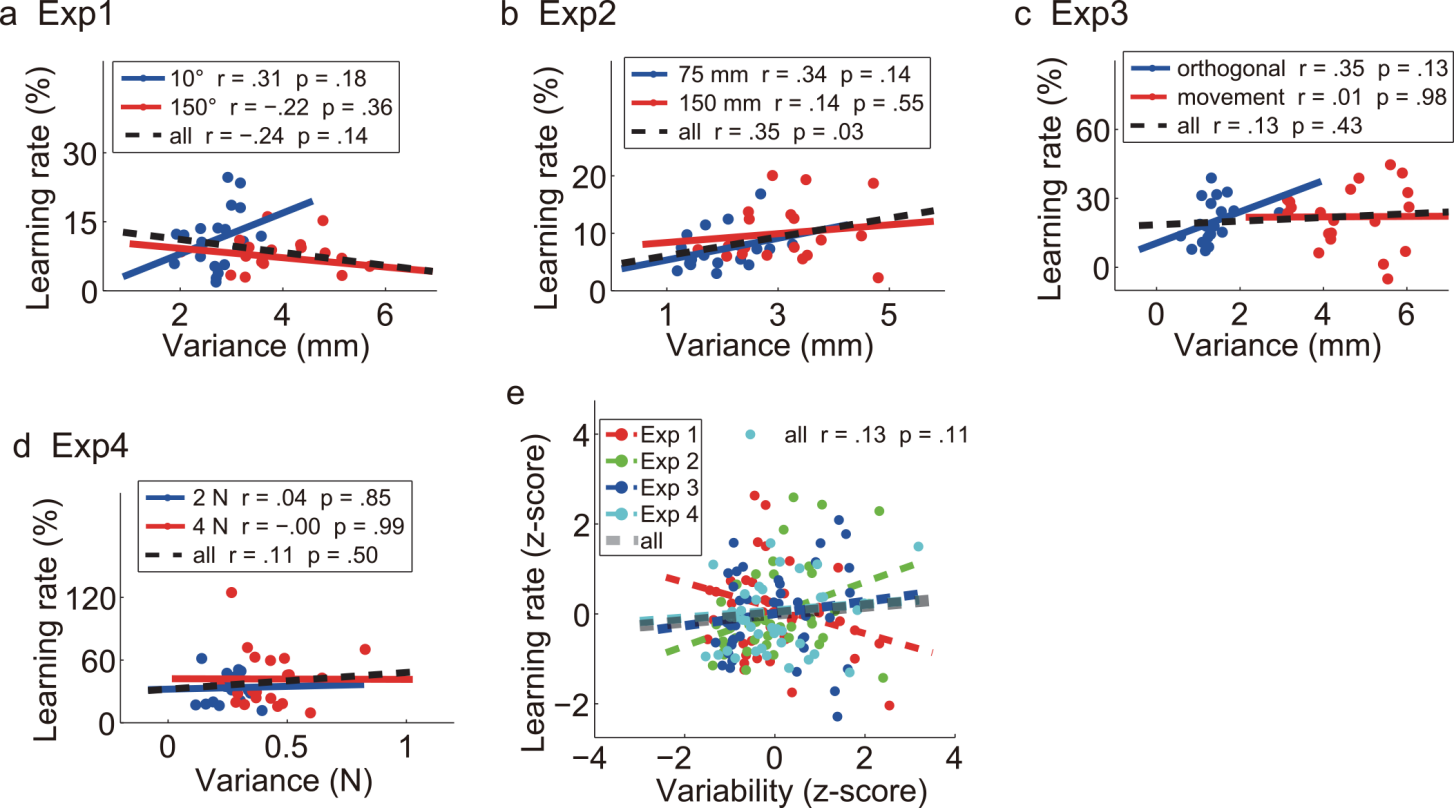
**Correlation analysis based on inter-individual difference for Exp1-4 (a-d) and overall data set (e).** Each dot denotes an individual’s average data. Within-condition and across-condition correlations are shown separately. For overall data set, the data was normalized as z-scores for each experiment separately and then pooled together.

Note that the above analyses used within-subjects comparisons. We also performed correlational analysis on inter-individual differences to search for possible correlations between variability and learning rate. For each experiment, correlation analysis (within a condition or across two conditions) failed to find a consistent positive correlation between variability and learning rate (Fig 6). The only significant result was from pooled data of two conditions in Exp2 (*p* = .03). We also normalized data across conditions for correlation calculation and none of experiments returned significant result. After normalization, the correlation analyses yielded *r* = .05, .24, .02, .28 and *p* = .77, .13, .90, .10 for Exp1-4, respectively. Correlation analysis based on normalized data across experiments also found that variability cannot predict individual’s learning rate (Fig 6E). Thus, our data suggests that there is no simple, positive relation between variability and learning rate.

The visual uncertainty for locating a cursor was measured by the control experiment, which indicated that uncertainty was higher for the 150° target than for the 10° target (*p* < .05, Fig 5E). The values of σ from fitted psychometric functions were 1.63±0.14mm versus 1.41±0.09mm for these two targets, respectively.

### Results from meta-analysis

**Fig 7:**
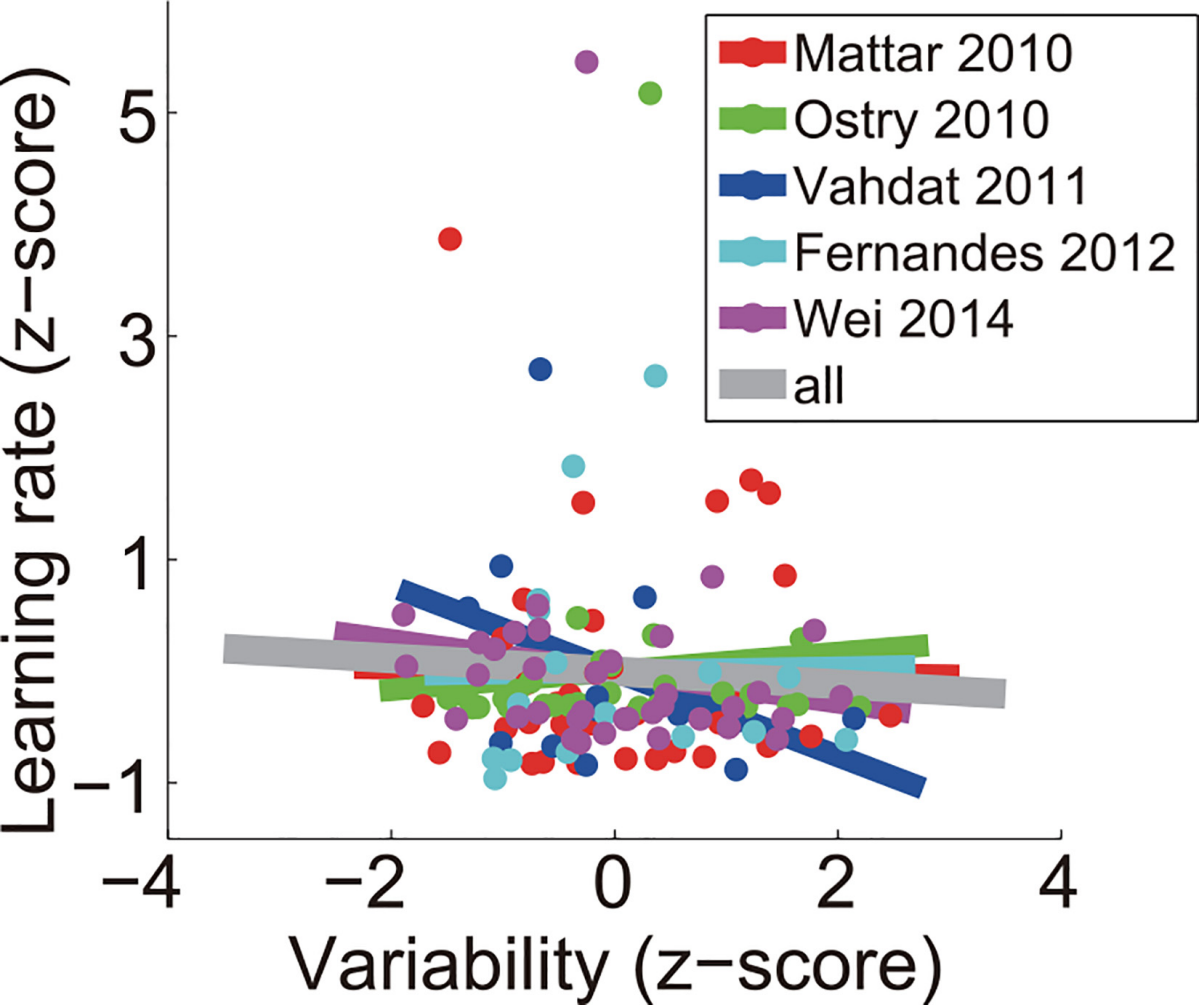
Results from a meta-analysis of 5 independent motor adaptation studies (n = 132). There is no significant correlation for each study (*p* = .94, .66, .20, .95 & .41 for 5 studies, respectively) and for overall data (*r* = -.058, *p* = .51). Each dot is from a single subject; different studies are labeled with different colors. Regression lines are shown for individual studies (color) and for the overall data set (gray)**. **

We searched for possible correlation between variability and learning rate in existing studies (Fig 7). Specifically, we performed a meta-analysis on existing data sets that tested motor adaptation. They involved commonly used perturbations, including visuomotor rotation [51], visuomotor gain [52], and velocity-dependent force fields [53–55].

None of these studies exhibited significant correlation between learning rate and variability (Mattar and Ostry, *r* = .013, *p* = .94; Ostry et al., *r* = -.084, *p* = .66; Vahdat et al., *r* = -.38, *p* = .20; Fernandes et al., *r* = -.017, *p* = .95; Wei et al., *r* = -.14, *p* = .41). In fact, all correlation coefficients except one were negative (Fig 7). Even when pooling all the data (n = 132), we still did not find significant correlation (Pearson *r* = -.058, *p* = .51; Spearman *r* = -.081, *p* = .36). If the correlation was at least r = .17, which implied a practically irrelevant R^2^ of .03, we would have had a 95% probability of observing it. So the correlation between variability and learning rate did not exist or was so small that it was practically irrelevant.

### Power analysis for the null results

We reported 4 null results to support our theoretical claims. They included the differences of learning rate between variability conditions in Exp3 and Exp4, the correlation between variability and learning rate for our data and for the meta-analysis. To estimate how reliable these null results were, we performed standard permutation tests to reshuffle the original data sets and recalculated the dependent measures (the rate difference Δ and correlation coefficient *r*). Using permutation 10000 times, we approximated the distributions of these dependent variables and then calculated how much larger these observed effect should become to obtain a significant result (i.e., against our null results with *α* = 0.05 & power = 0.8). The rate difference Δ should increase from 2.3% (Cohen’s d = 0.11) to 8.0% and from 8.3% (Cohen’s d = 0.33) to 14.9% for Exp3 and Exp4, respectively. The *r* should increase from 0.088 to 0.181 and from -0.058 to 0.204 for our data and meta-analysis data, respectively. These results mean that the obtained effect size should be 3.5, 1.8 and 2.1 times larger in order to reach significant levels for the first three tests. For the last null result, the correlation coefficient will need to change sign, which is very unlikely. Furthermore, we calculated how many participants were needed to obtain significant correlation results based on the effect sizes reported by Wu et al’s study. For correlation coefficients of 0.76 and 0.45 as shown in their experiment 1 and 2, the minimum numbers of subject were 11 and 34, respectively, in order to detect a significant correlation with a power of 0.8. In our study, we recruited 20 subjects for each experiment and they were repeatedly measured for two conditions. For meta-analysis, we had a total of 132 subjects. Thus, we had decent power to observe the effect if it existed (see Fig 6). We thus think our null results are reliable with sufficient power.

## Discussion

Using a trial-by-trial adaptation paradigm, we found that the rate of learning is independent from baseline variability. Larger variability was associated with slower, faster or similar learning rates in our four experiments, even though they all examined adaptation to visual perturbations. Furthermore, we found that inter-individual difference in baseline variability could not reliably predict learning rate, both in our own data and previously published studies spanning from force field learning to visuomotor learning. Our simple Kalman filter model, which has been shown to be a useful model for motor adaptation, predicts that different computational factors simultaneously affect learning rate and motor variability. The model simulations also suggest that the relation between variability and learning rate varies widely in motor adaptation, possibly depending on which computational factor dominates the performance in a specific task.

The seemingly divergent results in our four experiments can be qualitatively explained by taking into account sensory uncertainty or observation noise (model prediction 1, Fig 3A). Previous studies found that perturbations with smaller observation noise led to faster learning [37,40,41]. For our four experiments, subjects had to estimate movement error induced by visual perturbations in order to compensate for it in the next trial. For this estimation, the nervous system needs to combine the visual feedback with feedback from other modalities, including proprioceptive cues (for reaching in Exp1-3) and non-visual force cues (for force production in Exp4). This is a typical cue combination scenario, where Bayesian statistics have successfully explained a variety of findings. According to Bayesian cue combination theory, how much a visual perturbation can bias the final estimate is a function of its relative sensory uncertainty to other sensory cues. Higher uncertainty with visual error leads to a smaller bias, which in turn leads to slower learning.

In Exp1, visual uncertainty about localizing a cursor was higher with more depth as evidenced by our 2AFC results from the Control experiment (Fig 5E). This, as predicted by sensory uncertainty, will lead to the observed slower learning. For Exp2 with two different reaching distances, it has been shown that precision of the proprioceptive position sense is reduced with larger reaching distances [36]. Thus the longer reach (150 mm) is associated with more proprioceptive uncertainty. As shown in Exp2, the learning percentage to visual perturbations is less than 10%, suggesting that proprioception plays a dominant role in localizing the reaching endpoint in the trial-by-trial paradigm. Thus, decreased precision in proprioception with the larger reach distance (and thus relatively better precision in visual localization) leads to faster learning. For Exp3, visual perturbations in the two directions are shown in close vicinity, thus their sensory uncertainty is similar. The proprioceptive uncertainty is also similar since reach distance and direction are identical for these two conditions. Their corresponding adaptation rates are thus expected to be similar, in accordance with our data.

For force estimation in Exp4, non-visual cues such as signals from receptors in muscle spindles and tendons are associated with substantial uncertainties as compared to visual cues. Previous psychophysical studies found that without vision human’s force discrimination was very imprecise, with just noticeable difference (JND) being roughly 7% of the reference force [56,57]. Meanwhile, visual uncertainty of force feedback in our experiment was considerably smaller. The smallest visual perturbation (for a 0.3N perturbation) corresponded to a displacement of 72 pixels on the monitor, which was substantially larger than the JND in vision [58]. In fact, non-visual cues are so unreliable as compared to visual ones that people are unable to detect a distortion of visual representation of force until the distortion amounts to 36% of the target force [59]. This means that even the largest visual perturbation used in Exp4 was below the non-visual force detection threshold (30% and 15% of the target force in the 2N and 4N condition, respectively). Thus here we can reasonably assume that visual cues dominate the perception of force, similar to visual capture phenomenon in visuo-auditory illusions [60,61]. Hence, in Exp4 visual uncertainty is considerably smaller than uncertainty of non-visual cues and it is also similar between two force conditions. As a result, two force conditions should have similar adaptation rates, consistent with our findings.

There are many kinds of noise and uncertainty and it is important to acknowledge this diversity. In fact, for the four experiments presented here, the difference of movement variability between conditions are largely determined by execution noise, while the difference of the learning rates appear to be affected by sensory uncertainty. Therefore, these findings provide strong indication that the relation between movement variability and learning rate is task-specific. Our model simulations also highlight that it makes no computational sense for movement variability to *generally* increase learning speed. In fact, our simulation and new data reveal that increased variability does not typically enable faster learning and sometimes even slows it down.

Our proposition does not preclude a possible positive correlation between motor variability and learning rate. In fact, it has been proposed that variability is important for exploration during skill acquisition [22,62]. Skill acquisition tasks are typically novel to the participant and demand a period of time to learn the task requirements. For instance, Sternad and colleagues investigated a novel throw task which required participants to explore the solution space spanned by two control variables [3]. Over repeated trials, people converge to solutions where inevitable motor variability minimally impacts the movement accuracy.

Wu et al.’s study went one step further by suggesting that initial variability can predict the learning rate in both reinforcement learning tasks and motor adaptation tasks [26]. It was the first study to show the facilitatory effect for error-based motor adaptation. The authors suggested that: “… motor exploration provides information that is useful for improving the fidelity of the internal representation of the gradient function and the confidence in that representation.” As their adaptation task involves learning to reach in complex force fields, we speculate that its solution space is relatively complicated as compared to that of our simple trial-by-trial perturbation tasks. Thus, similar to acquiring a novel motor skill, individuals might need to first explore the solution space during initial learning and before fine-tuning their control strategy. During this process, initial motor variability may enable people to better explore the solution space and facilitate the convergence to a solution, as manifested by faster learning. This can be a plausible explanation for Wu et al.’s findings about error-based adaptation. However, our findings suggest that this facilitation effect of variability does not generally hold, particularly for our simple motor adaptation paradigms where exploration is of little importance.

An unlikely account for the difference between our findings and those of Wu et al.’s is that variability was defined and quantified in different ways. Wu et al.’s study used force field perturbations and measured the lateral force profile in so-called error-clamp trials. This baseline force profile was projected onto the ideal velocity-dependent force patterns and the variance of the resulting profile was regarded as task-relevant variability. Thus, though not explicitly stated in their paper, task-relevant variability appears to be defined as movement variability in the direction of applied perturbations. Our study uses visual endpoint perturbations and the performance measure is also the endpoint error, a typical error measure for this type of adaptation. As we quantify the variance of endpoint in the direction of applied perturbation only, this variability is similarly task-relevant. Given the high-dimensional nature of movement data, it is highly probable to find positive correlations between variability and learning rate when searching for variability measures. However, we submit that other variability measures are relevant for the learning tasks examined here.

For trial-by-trial adaptation examined here, intermittent visual feedback is vital for good performance as people make adaptive changes to visual perturbations. As a result, sensory uncertainty emerges as a determinant factor for learning. This finding is not new, though. Other studies, including our previous work, indicate that artificially increasing visual uncertainty of error feedback leads to lower adaptation rate in reaching tasks [37,40]. However, the present study provides two new insights: first, the effect exists for kinematic tasks (i.e., reaching) as well as for kinetic tasks (force production); second, the effect exists even when sensory uncertainty increases naturally for the same task that is executed differently (i.e., reaching to a direction with more depth), in contrast to previously investigated situations when sensory uncertainty is experimentally inflated (i.e., blurring the visual feedback).

The important aspect of this work is that it shows that the relation between variability and the speed of motor learning is far more complicated than appreciated by previous studies. Notably, our study only focuses on error-based motor learning and we expect that the relation may be even more complicated if other types of motor learning are considered. Lastly, our findings are supported through a range of experiments and a broad meta-analysis. Wherever possible, such meta-analyses should become standard in the movement literature before claiming a high level of generality for any effect.
